# Eighty-Four-Month Clinical Outcomes of Autologous Dentin Graft Using Tooth Transformer^®^ and Concentrated Growth Factors in Maxillary Atrophy: A Retrospective Study of 31 Patients

**DOI:** 10.3390/jfb16100357

**Published:** 2025-09-23

**Authors:** Gianna Dipalma, Alessio Danilo Inchingolo, Francesca Calò, Rosalba Lagioia, Paola Bassi, Elisabetta de Ruvo, Francesco Inchingolo, Andrea Palermo, Grazia Marinelli, Angelo Michele Inchingolo

**Affiliations:** 1 Department of Interdisciplinary Medicine, University of Bari “Aldo Moro”, 70124 Bari, Italy; gianna.dipalma@uniba.it (G.D.); francesca.calo@uniba.it (F.C.); rosalba.lagioia@uniba.it (R.L.); paola.bassi@uniba.it (P.B.); studio.deruvo@libero.it (E.d.R.); graziamarinelli@live.it (G.M.); angelomichele.inchingolo@uniba.it (A.M.I.); 2 Department of Biomedical, Surgical and Dental Sciences, Milan University, 20122 Milan, Italy; 3 Department of Experimental Medicine, University of Salento, 73100 Lecce, Italy; andrea.palermo@unisalento.it

**Keywords:** Tooth Transformer^®^, Medifuge MF200^®^, autologous tooth, biomaterials, concentrated growth factors, bone remodeling, autologous graft, tooth graft, sinus lift, sticky bone, osseointegration, maxillary atrophy, regenerative implantology

## Abstract

Aim: This retrospective observational clinical cohort study evaluated 84-month clinical and radiographic outcomes of a regenerative protocol combining autologous dentin grafts processed with the Tooth Transformer^®^ device and Concentrated Growth Factors (CGFs) in patients with severe maxillary atrophy undergoing sinus augmentation with simultaneous implant placement. Materials and Methods: Thirty-one patients (30–75 years) with residual crestal bone height ≥ 5 mm and requiring extraction of ≥2 molars were included. Extracted teeth were processed with the Tooth Transformer^®^ to obtain demineralized dentin granules (500–1000 µm), which were combined with CGFs prepared using the Medifuge MF200^®^ to form “sticky bone.” All patients underwent sinus lift via a lateral window approach (Hilt Tatum technique) with simultaneous placement of 98 implants (12–14 mm), which were loaded after six months. Results: At the 84-month follow-up, no implant failures or peri-implantitis were recorded. CBCT and clinical evaluations showed stable regenerated bone volume and absence of peri-implant bone resorption. All patients received fixed prostheses within six months without complications. Conclusions: The combined use of processed autologous dentin and CGFs proved to be a safe, predictable, and effective regenerative technique in cases of severe maxillary atrophy, with a 100% implant survival rate at five years.

## 1. Introduction

Severe maxillary atrophies represent a major clinical challenge in implantology and prosthodontics, often necessitating complex regenerative procedures prior to the placement of osseointegrated implants [1,2,3,4,5]. Alveolar bone loss, caused by dental extractions, prolonged edentulousness, trauma, or periodontal disease, compromises the volume and quality of residual bone, hindering implant rehabilitation in the absence of adequate bone regeneration [6,7,8,9,10].

In recent decades, regenerative medicine has made important advances, moving toward increasingly effective and personalized biological solutions, thus accelerating the remodeling of newly formed bone [11,12,13,14,15,16]. Traditional graft materials (autologous, homologous, heterologous, or synthetic) have many limitations, including donor site morbidity, possibility of resorption, high cost, and variability in biological response [17,18,19,20]. Additionally, the literature reports specific complications for each category: donor-site morbidity and resorption for autografts, immunological risk and variable biological response for allografts, persistence of material and foreign body reactions for xenografts, and inadequate resorption or mechanical instability for synthetic materials. Common adverse events include infections, wound dehiscence, graft exposure, and regenerative failure, with overall complication rates reported up to 20% depending on the material and procedure. The literature has shown that, at the histologic level, these biomaterials do not generate 100% neoformed bone, but about 50% in the long term, that is, after 24–30 months [21,22,23,24]. However, these biomaterials ensure granule stability, thus providing effective osteoconduction [25,26,27,28,29].

In this context, materials of autologous origin, particularly autologous tooth, have emerged as a promising alternative due to their biocompatibility, absence of immune reactions, and reduced infectious risk [30,31,32,33,34,35]. Histologic literature has shown that more than 80% of newly formed bone is present at 12 months with these materials [36,37,38].

One of the most innovative solutions is the use of autologous dentin as a graft material [39,40,41,42,43]. Properly processed, dentin has biochemical characteristics similar to those of bone, including an inorganic component based on hydroxyapatite (about 61%) and an organic component consisting mainly of type I collagen and bioactive proteins such as BMPs, osteopontin, and the growth factors IGF and TGF-β [39,40,41,42,43]. These characteristics give dentin tissue osteoinductive and osteoconductive properties of considerable interest for bone regeneration [44,45,46,47,48].

The Tooth Transformer^®^ device enables the transformation of a patient’s extracted teeth into a granular, sterile, biocompatible graft material through an automated process of decontamination, demineralization, and preservation of bioactive factors [49,50,51,52]. The final product is characterized by an ideal architecture (with 500–1000 μm granules), suitable for promoting the adhesion and migration of osteoprogenitor cells, as well as the formation of a vascular network essential for regenerative success [53,54,55,56].

In parallel, the use of Concentrated Growth Factors (CGFs) has revolutionized the approach to regenerative surgery [57,58,59,60,61,62]. CGFs are obtained by centrifugation of a patient’s peripheral venous blood, isolating a fibrin matrix highly concentrated in growth factors such as PDGF, TGF-β1, VEGF and IGF, as well as mesenchymal stem cells and leukocytes [63,64,65,66]. Compared with earlier formulations such as PRP and PRF, CGFs offer greater structural stability, longer cytokine release, and better intraoperative handling [67,68,69,70]. Their key role is to stimulate cell proliferation, angiogenesis and anti-inflammatory action, thus helping to accelerate hard and soft tissue regeneration [71,72,73,74].

The synergistic combination of autologous dentin and CGFs has led to the development of an advanced biological graft, also known as “*Sticky Bone*”. This compound is moldable, cohesive, stable and rich in bioactive factors, capable of acting simultaneously as an osteoconductive scaffold and as a stimulator of osteogenic activity [75,76,77,78,79,80]. This approach avoids bone harvesting from intra- or extraoral donor sites, reducing surgical time, postoperative morbidity, and patient discomfort, in line with the principles of personalized medicine and tissue engineering [81,82,83,84].

Although the efficacy of both components has already been demonstrated in various clinical settings (alveolar preservation, sinus lift, minor bone defects), little scientific evidence is available on the combined use of autologous processed dentin and CGFs in cases of severe maxillary atrophy with long-term follow-up [85,86,87].

The purpose of the present retrospective study was to evaluate the clinical and radiographic outcome with CBCT, at 84 months of follow-up, of 31 patients with severe maxillary atrophy all treated with the same technique [88,89,90]. Autologous dentin treated with Tooth Transformer^®^ and Concentrated Growth Factors (CGFs) was used in all patients, applying the method through the use of the Medifuge 200^®^ centrifuge, with the aim of accelerating peri-implant bone regeneration. The objective was to test whether this protocol allows sufficient increase in bone volume and density to be achieved quickly (within six months) to be able to load fixed prostheses [91,92,93,94,95,96]. This technique has been shown to be feasible, predictable, and durable [97,98].

## 2. Materials and Methods

### 2.1. Ethical Considerations

The study was conducted by the Declaration of Helsinki, and approved by the Institutional Review Board of the University of Bari “Aldo Moro” (Local Ethics Committee Secretariat. Prot. 1979/CE, approval date 16 December 2024). 

### 2.2. Study Design

Thirty-one patients (aged 30–75 years) with severe maxillary atrophy and residual crestal bone height ≥ 5 mm were enrolled according to strict inclusion and exclusion criteria. At least two molars were extracted in each patient to obtain ≥4 g of autologous dentin, which was mechanically cleaned, fragmented, and demineralized using the Tooth Transformer^®^, yielding dentin granules (500–1000 µm) rich in collagen and BMP-2. In parallel, autologous blood was centrifuged with the Medifuge MF200^®^ to obtain CGFs. The dentin granules were combined with liquid AFG and small fragments of CGF clots to produce “sticky bone” a cohesive matrix used for grafting. All patients underwent lateral window sinus lift (Hilt Tatum technique), where sticky bone was placed together with CGF membranes, and 98 endosseous implants (12–14 mm) were simultaneously inserted. Prosthetic loading occurred after six months.

Inclusion criteria:

- Age between 30 and 75 years (The selected age range of 30 to 75 years represents the population commonly considered for implant rehabilitation procedures with maxillary atrophy because it has a sufficiently stable bone metabolism to support regenerative processes, minimizing the confounding variables associated with very young patients (still in the bone development phase) or very elderly patients (with advanced osteoporosis or multiple comorbidities).
- Residual crestal bone height of 5 mm or more.
- Need for extraction of at least two molars for severe periodontal reasons to ensure a minimum amount of 4 g of available autologous dentin.
- Severe maxillary atrophy candidates for sinus lift and concomitant implant placement.
- Adequate oral hygiene status (Full-Mouth Plaque Score < 20%).
- Non-Smoker or light smoker (<10 cigarettes/day).

Exclusion criteria:

- Patients with severe systemic diseases that may interfere with the bone regeneration process including: uncontrolled diabetes mellitus (HbA1c > 7.5%), cardiovascular disease, neoplasms undergoing treatment, advanced chronic liver or kidney disease (stage ≥ 3).
- Infectious or autoimmune diseases (systemic lupus erythematosus, rheumatoid arthritis).
- Amount of autologous dentin less than 2 g or no need for dental avulsions to ensure adequate availability of useful biomaterial for bone regeneration.
- Other factors that could compromise bone regeneration or implant osseointegration (patients undergoing immunosuppressive therapy or intravenous bisphosphonates).

### 2.3. Preparation of the “Sticky Bone”

**Figure 1:**
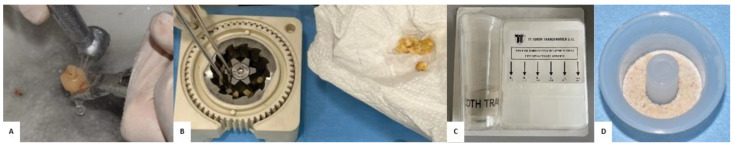
Preparation of autologous dentin. (**A**) The extracted tooth is cleaned with the use of a diamond bur mounted on a turbine to remove decay, tartar, filling materials and the dental pulp. (**B**) Dental fragments are placed in the Tooth Grinder^®^. (**C**) The demineralization kit and cylinder for exhausted liquids. (**D**) Demineralized autologous dentin granules.

After extraction, each extracted tooth was thoroughly cleaned by mechanical removal of tartar, carious tissue, dental pulp and any pre-existing restorative materials such as composite or amalgam (Figure 1). Once this cleaning step was completed, the tooth was reduced into fragments ranging in size from 0.3 to 0.6 mm. The resulting fragments were placed inside the Tooth Grinder^®^ (TT Tooth Transformer s.r.l. via Washington, 59, 20146 Milan, Italy), which is a component of the Tooth Transformer^®^ system (TT Tooth Transformer s.r.l. via Washington, 59, 20146 Milan, Italy) that allows tooth fragments to be shredded. The Tooth Grinder^®^ is made of heat-resistant plastic and equipped with medical steel blades, is fully autoclavable, and is designed to safely process dental tissue in a standardized manner.

**Figure 2:**
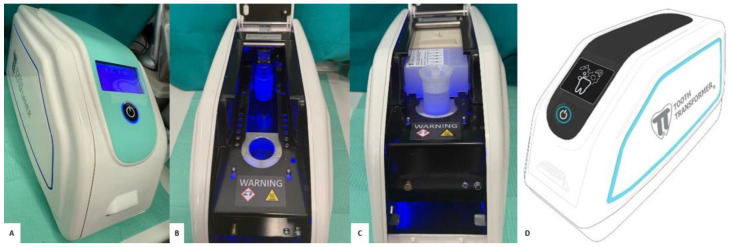
(**A**) Tooth Transformer^®^. (**B**) Device open and not loaded. (**C**) Device loaded with Tooth Grinder^®^ and accessory kit. (**D**) Picture of Tooth Transformer^®^ device.

The Tooth Transformer^®^ is capable of performing, in an automated manner, a sequence of actions including fragmentation, chemical-thermal decontamination, removal of inorganic components, and demineralization of dentin (Figure 2). Using a disposable accessory kit, the granules underwent a process of demineralization, disinfection, and rinsing. The kit was provided by TT Tooth Transformer s.r.l. via Washington, 59, 20146 Milan, Italy. This kit included a cartridge containing six liquid solutions: two of them consisted of hydrochloric acid (0.1 M) and H_2_O_2_ (10 percent), one contained demineralized H_2_O, and the other four were mineralized H_2_O solutions, which were used in four separate steps of the process to remove acidic residues (Figure 1C). After about 25 min of processing, a demineralized dentin granule rich in collagen, bone morphogenetic protein (BMP-2) and other growth factors with osteoinductive potential was obtained. The resulting dentin granules range in size from 500 to 1000 µm, which are ideal for promoting osteoblast adhesion and proliferation by proteins such as BMP-2 and TGF-β. At the end of the treatment, the resulting granules were collected in a basket, while the spent fluids were conveyed into a special cylinder.

**Figure 3:**
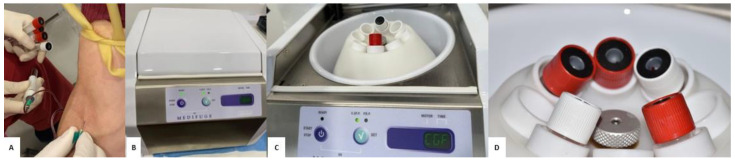
(**A**) Peripheral venous blood sampling. (**B**–**D**) Medifuge MF200^®^ (Silfradent, Santa Sofia, Italia).

Parallel to the preparation of dentin material, peripheral venous blood was drawn from the patient for the production of autologous platelet concentrates using the Medifuge MF200^®^ system (Silfradent, Santa Sofia, Italy). This is an advanced medical device designed to separate blood components in order to obtain Concentrated Growth Factors (CGFs), which is an autologous fibrin matrix with a high concentration of growth factors. This instrument allows simultaneous processing of up to eight tubes, also organized in groups of two, making it particularly suitable for clinical use in areas such as oral surgery, regenerative orthopedics and aesthetic medicine (Figure 3).

The centrifugal system incorporates an innovative rotor with automatic ventilation, which maintains a constant internal temperature throughout the entire processing cycle, thus preventing overheating of the sample and preserving its biochemical integrity. The materials used in the manufacture of the components in contact with the sample, such as the tube jackets and the rotor itself, are heat-resistant, antistatic, easily cleaned and compatible with autoclave sterilization up to 135 °C, thus ensuring maximum hygiene and reusability in medical environments.

**Figure 4:**
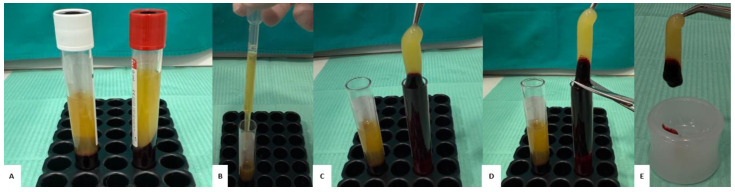
(**A**) White test tube and red test tube. (**B**) The AFG (Autologous Fibrin Glue) obtained from the white tube. (**C**–**E**) The fibrin clot resulting from the red tube.

An additional level of microbiological safety is provided by the built-in decontamination system, which uses reflected UVC light to sanitize the internal surfaces of the device between cycles. The operating cycle lasts five minutes, during which the rotor reaches a constant speed of 1000 rpm. This stability is ensured by a microprocessor-based electronic control system, which automatically adjusts the rotation speed, avoiding fluctuations or variations that could compromise the quality of the material obtained. The internal motor requires no routine maintenance, and the noise level of the device remains below 57 dBA, resulting in compliance with acoustic comfort standards required in clinical settings. Depending on the type of tube used, different blood derivatives can be obtained (Figure 4). White tubes, characterized by a smooth surface and free of anticoagulants, allow separation of the liquid component called AFG (Autologous Fibrin Glue), a fluid protein matrix rich in thrombin and growth factors. Red tubes, with a rough inner surface, promote the formation of a three-dimensional clot known in the literature as a “fibrin clot” or “CGF clot” consisting of a yellow part, composed mainly of fibrin, and a red part, rich in red blood cells and CD34+ cells. The size and firmness of the clot depend on the individual platelet concentration. Finally, green tubes, containing heparin, allow obtaining a plasma rich in cytokines and soluble factors that, while remaining in liquid form, can be used as an adjuvant in regenerative applications.

**Figure 5:**
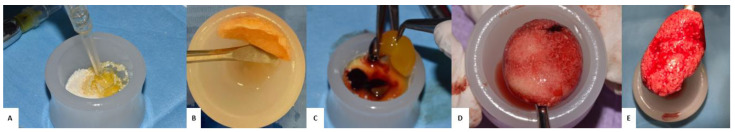
Preparation of Sticky Bone. (**A**,**B**) AFG is mixed with demineralized dentin granulate. (**C**) A small fragment of CGFs clot is added. (**D**,**E**) Sticky Bone.

To obtain “*sticky bone*”, demineralized dentin granulate was mixed with AFG taken from the white tubes (Figure 5). The main advantage of using the white tube lies in the fact that, immediately after centrifugation, the CGF (Concentrated Growth Factor) obtained from it is in the liquid phase, maintaining this state for at least 12 min. During this time interval, the granulate resulting from the transformation of the autologous tooth using the Tooth Transformer^®^ device, with a size between 500 and 1000 µm, is introduced inside a dappen and mixed with the liquid component taken from the white tube. The resulting mixture is allowed to polymerize for about 12 min, during which time it takes on a cohesive and easily manipulated consistency in the form of a gel. This process allows effective compaction of the granules obtained from grinding the autologous tooth. To further promote cohesion, increase the stability of the compound and enrich it with CD34+ protein, contained in the red fraction of CGF, a small fragment of the CGF clot was added. The latter, rich in fibrin and hematopoietic stem cells, was obtained from the red end component of a fibrin gel centrifuged in a red tube; this portion is sectioned and then mixed with the liquid gel from the white tube.

The final product, termed sticky bone, consists of the compaction of dentin granules into a gelatinous matrix rich in growth factors. This cohesive structure facilitates graft shaping, improves stability and integration of the granules, and allows sustained release of bioactive factors, promoting angiogenesis and bone regenerative processes.

### 2.4. Surgical Procedure

**Figure 6:**
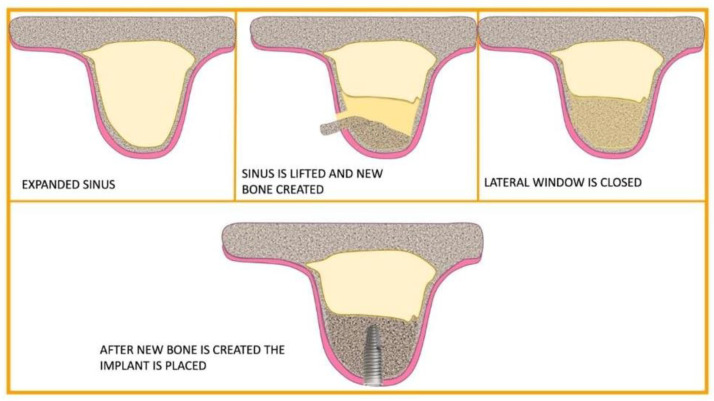
Sinus lift technique according to Hilt Tatum.

All patients underwent sinus lift surgery by lateral approach, according to the technique described by Hilt Tatum (Figure 6).

**Figure 7:**
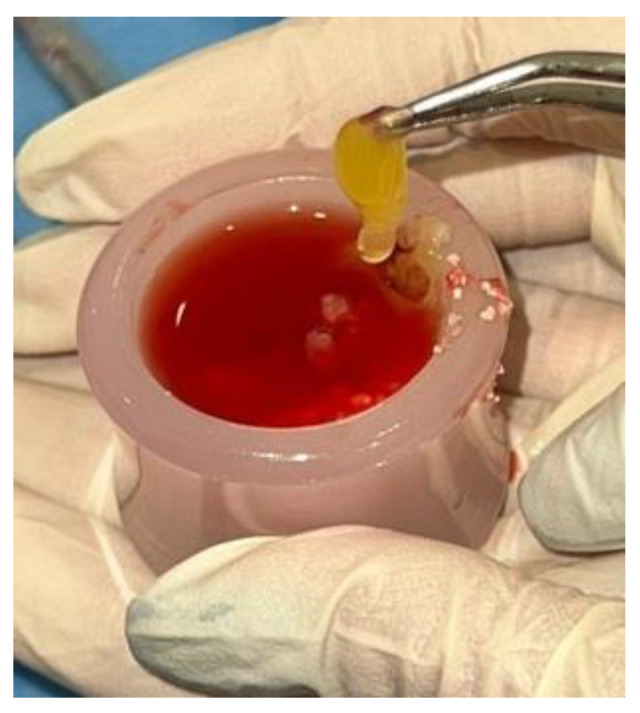
Biological membrane of CGF.

After incision and lifting of the mucoperiosteal flap, a bony window in the lateral wall of the maxillary sinus was traced and osteotomized, then lifted to expose Schneider’s membrane. The latter was gently peeled back and raised, creating the space necessary for the insertion of the graft material. At the base of the resulting bone cavity, the CGF clot gel (5–6 mm) was placed, followed by the insertion of sticky bone obtained by mixing demineralized dentin and AFG. Finally, a third CGF clot was pressed and shaped to form a biological membrane covering the graft, protecting the regenerative site, before repositioning and fixation of the bone hatch (Figure 7).

At the same time as the regenerative procedure was performed, 98 endosseous implants were placed in the 31 patients with a minimum length of 12–14 mm. Implant placement was made possible by the presence of a minimum residual bone ridge of at least 5 mm in all patients, which was a fundamental requirement to ensure primary stability. The use of sticky bone allowed for a vertical bone augmentation of about 8 mm, achieving an overall bone height of 13–15 mm at the end of treatment. The implants underwent prosthetic loading after a six-month healing period. All implants at the prosthetic stage had a primary stability of 70 Newtons as measured by ISQ (Implant Stability Quotient).

### 2.5. Clinical and Radiographic Evaluation

The effectiveness of the regenerative protocol was evaluated clinically and by Cone Beam Computed Tomography (CBCT) at T0 (preoperative), T1 at 6 months (prosthetic loading) and T2 at 84 months (long-term follow-up). Implant survival, bone volume stability and absence of complications were evaluated.

## 3. Results

### 3.1. CBCT Radiographic Evaluation at T0, T1 and T2

The initial crestal bone height, as assessed by CBCT, was always 5 mm or more, a necessary condition to ensure primary stability of the implants during the concomitant sinus lift. After surgery, the total bone height achieved ranged from 13 to 15 mm, with an estimated average increase of about 8 to 10 mm, achieved by application of the combined sticky bone technique (Tooth Transformer^®^ + AFG) and CGFs. CBCTs at 6 months and 84 months showed stability of the regenerate, with no significant resorption and maintenance of peri-implant bone volume.

CBCT analyses showed:

Preoperative bone height (T0): always ≥5 mm;

Bone height at 6 months (T1): estimated mean increase of ~ 8.6 mm with final height between 13 and 15 mm;

Bone height at 84 months (T2): stable bone volume, negligible mean decrease (<0.5 mm), i.e., suggesting maintenance of long-term bone regeneration-layer based on observational findings.

Densitometric analysis suggests that the newly formed tissue had density values compatible with mature bone (estimated range: 700–850 Hounsfield Units), confirming the regenerative efficacy of the Tooth Transformer^®^ + CGFs combination.

### 3.2. Implant Survival, Stability and Complications

During the average follow-up of 84 months, all 98 inserted implants remained fully functional, with no failures. Implant survival was 100%. No intra- or postoperative complications emerged: no cases of surgical dehiscence, infection, implant mobility or peri-implantitis were observed. Primary stability of the implants was always ensured by ≥ 5 mm residual bone ridge, and all patients received fixed prostheses within six months after surgery according to the standard protocol.

In addition, radiographic follow-up confirmed maintenance of marginal bone levels without detectable peri-implant bone loss beyond the negligible changes reported (<0.5 mm on CBCT). Patient-reported outcomes were not systematically collected; however, no patients reported discomfort, pain, or functional limitations during the follow-up period.

All implants had primary stability at the time of insertion due to ≥5 mm residual bone ridge. The mean ISQ value at loading stage (6 months) was 70 Ncm or more, indicating effective osseointegration. No implants showed loss of stability during follow-up. These data, together with the absence of stability loss, confirm the effectiveness of the regenerative protocol.

### 3.3. Clinical Cases

#### 3.3.1. Case 1—Female Patient, 52 Years Old

**Figure 8:**
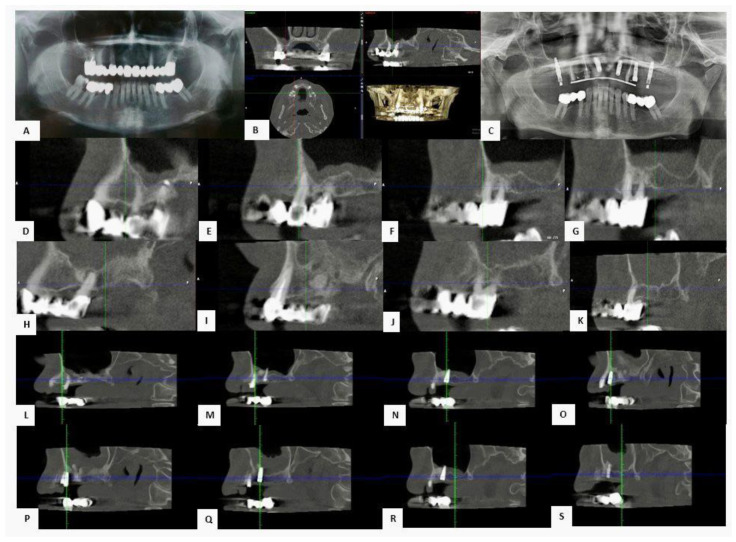
(**A**) Pre-operative OPT. (**B**) Pre-operative CBCT. (**C**) OPT check after 84 months. (**D**–**K**) Pre-operative CBCT sections of areas 1.4, 1.5, 1.6, 1.7, 2.4, 2.5, 2.6 and 2.7. (**L**–**S**) CBCT sections after 84 months of areas 1.4, 1.5, 1.6, 1.7, 2.4, 2.5, 2.6 and 2.7.

The patient, aged 52, came in for a consultation with both aesthetic and functional needs. During the initial evaluation, a careful objective analysis of the oral cavity was performed, followed by the collection of the necessary diagnostic data, including a complete medical history, extraoral (frontal and lateral) and intraoral photographs, orthopantomography of the dental arches, and cone beam computed tomography (CBCT) of the upper arch (Figure 8).

The clinical and radiological examination revealed the presence of a metal-ceramic prosthesis on natural teeth, including 1.6, 1.5, 1.3, 2.3, and 2.6. It was therefore decided to proceed with the surgical extraction of both elements 1.6 and 2.6 due to advanced periodontal disease. After administering local anesthesia with a vasoconstrictor (adrenaline), the teeth were extracted by dislocation with levers and avulsion with surgical forceps. The extracted roots were processed to obtain approximately 4 g of autologous dentin to be used as biomaterial.

At time zero (T0), CBCT showed a residual bone height of 5.4 mm. A lateral sinus lift was then performed using adhesive bone enriched with CGFs (concentrated growth factors), followed by simultaneous placement of implants in the posterior and anterior regions. Six months after surgery (T1), a new CBCT showed significant bone regeneration with an estimated increase in height and density of around 800 HU, indicative of good quality newly formed bone. Unfortunately, for economic reasons, after seven years the patient still has a temporary upper prosthesis reinforced with resin supported by implants.

Long-term follow-up, conducted 84 months after surgery (T2), confirmed the stability of the clinical result: bone height was maintained, with no signs of resorption, and the bone and peri-implant tissues were perfectly intact.

#### 3.3.2. Case 2—Female Patient, 60 Years Old

**Figure 9:**
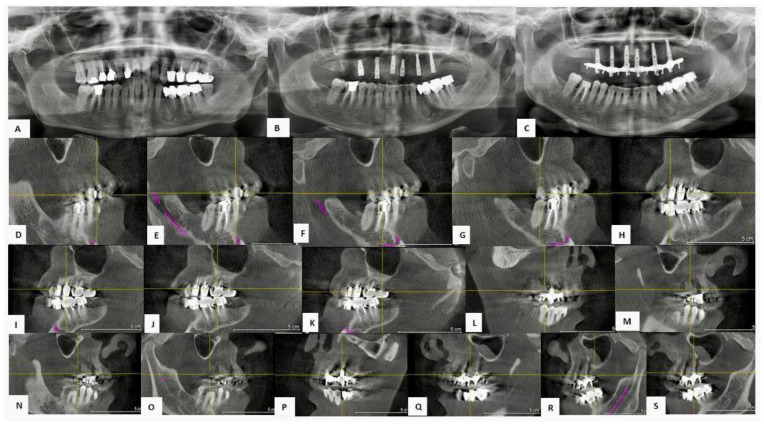
(**A**) Pre-operative OPT. (**B**) OPT check after sinus augmentation and after placement of six endosseous implants. (**C**) OPT check after 84 months. (**D**–**K**) Pre-operative CBCT sections of areas 1.4, 1.5, 1.6, 1.7, 2.4, 2.5, 2.6 and 2.7. (**L**–**S**) CBCT sections after 84 months of areas 1.4, 1.5, 1.6, 1.7, 2.4, 2.5, 2.6 and 2.7.

Following an initial objective assessment of the oral cavity and after careful analysis of the aesthetic and functional needs expressed by the patient, an appropriate diagnostic pathway was undertaken. This pathway included the collection of a complete medical history, the acquisition of both extraoral (in frontal and lateral projection) and intraoral photographic documentation, the performance of OPT of the dental arches and a CBCT of the upper jaw (Figure 9).

Joint analysis of the clinical and radiological data showed severe periodontal impairment of the upper dental elements, making their recuperation impossible. Therefore, it was decided to proceed with complete recuperation of the maxillary arch.

The surgical procedure involved atraumatic extraction of the remaining dental elements, followed by careful curettage of the post-extraction sites in order to remove inflammatory tissue or granulomatous in nature. Bone regeneration was then performed by a combined technique of using autologous demineralized dentin obtained with Tooth Transformer^®^ and CGFs. The Tooth Transformer^®^ provided, through processing of the extracted tooth elements, a fully autologous biomaterial with osteoinductive and osteoconductive properties. In parallel, the patient’s peripheric blood was subjected to centrifugation in order to obtain growth factor concentrates, including platelet-enriched fibrin and leukocytes.

Autologous demineralized dentin and liquid-phase CGF were mixed and placed into the remaining bone sites, with the aim of promoting tissue regeneration and accelerating bone healing processes. The surgical sites were covered with autologous fibrin membranes, also obtained by centrifugation of peripheral blood, and then sutured with resorbable threads.

In the same session, six endosseous implants were placed in the upper maxillary region, with lengths ranging from 12 to 14 mm, strategically placed to allow for future implant- supported prosthetic rehabilitation.

This combined technique achieved completely autologous and biologically active bone regeneration, helping to reduce healing time and minimize the incidence of postoperative complications.

#### 3.3.3. Case 3—Male Patient, 58 Years Old

**Figure 10:**
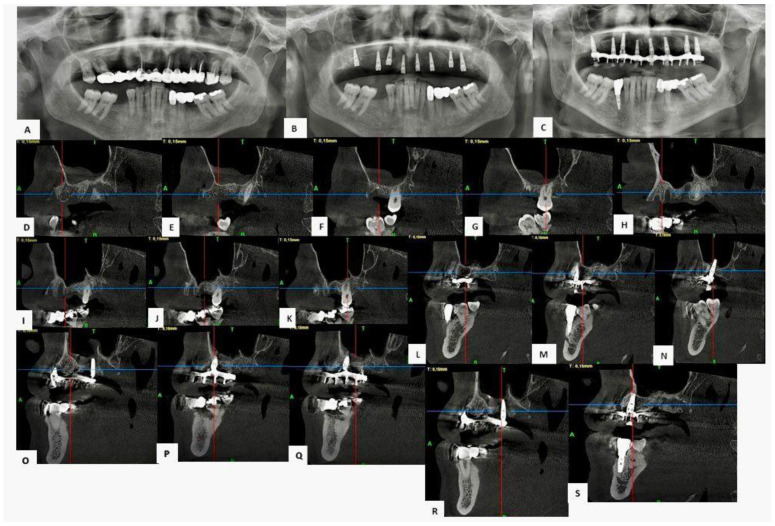
(**A**) Pre-operative OPT. (**B**) OPT check after placement of endosseous implants. (**C**) OPT check after 84 months. (**D**–**K**) Pre-operative CBCT sections of areas 1.4, 1.5, 1.6, 1.7, 2.4, 2.5, 2.6 and 2.7. (**L**–**S**) CBCT sections after 84 months of areas 1.4, 1.5, 1.6, 1.7, 2.4, 2.5, 2.6 and 2.7.

The 58-year-old patient underwent clinical examination and photo and radiographs (OPT and CBCT) (Figure 10). He presented with a complex clinical condition characterized by severe periodontal compromise and the presence of destructive caries on multiple dental elements of the upper jaw, specifically on teeth 1.3, 1.6, 1.7, 2.2, 2.3, 2.4, 2.5, 2.6, and 2.7. Reclamation of the upper oral cavity was performed by multiple extractions of the compromised elements, followed by careful curettage of the post-extraction areas in order to remove infected tissue and stimulate tissue healing.

Next, bone regeneration of the post-extractive sites in both the first quadrant and second quadrant was performed using a combined technique integrating the Tooth Transformer^®^ method with the application of CGFs. This strategy allowed to significantly improve the quality and quantity of regenerated bone, promoting a favorable environment for implant placement.

Implant planning was supported by radiological investigations using CBCT, performed before sinus lift, which allowed a detailed three-dimensional assessment of available bone volumes. Following sinus lift associated with overall bone augmentation, a volumetric increase of approximately 14 mm was achieved, making it feasible to place dental implants in optimal positions for chewing function and aesthetics. Contextually, endosseous implants with a variable length of 12–14 mm were placed.

The treatment demonstrated good clinical and radiographic success with a follow-up at 84 months, with adequate implant integration and excellent bone regeneration without obvious peri-implant complications.

## 4. Discussion

The results of the present study show that the use of a combined regenerative approach, based on the use of autologous demineralized dentin obtained through the Tooth Transformer^®^ system and platelet concentrates rich in growth factors (CGFs), represents an innovative and promising strategy for volumetric sinus augmentation and bone regeneration in the peri-implant site [99,100,101,102]. The clinical efficacy of the technique is confirmed by an implant survival rate of 100% over an average follow-up of 84 months, with no manifestations of intra- or postoperative complications [103,104,105,106,107,108].

An aspect of particular relevance concerns the vertical bone augmentation obtained, between 8 and 10 mm, and its stability over time, as documented by medium- and long-term tomographic (CBCT) investigations [109,110,111,112,113,114,115,116,117]. These data are in line with reports in the literature, which attribute osteoinductive properties to demineralized dentin due to the release of bioactive molecules such as bone morphogenetic proteins (BMPs) and collagen [118,119]. Supplementation with CGFs, known for their high concentration of biologically active growth factors, further enhances the regenerative process [120,121,122].

The presence of a residual bone ridge of at least 5 mm and the quality of the newly formed tissue allowed for optimal primary stability of the implants. ISQ values ≥ 70 detected at the time of functional loading confirm satisfactory osseointegration as well as implant consolidation, key elements to ensure long-term predictability of implant treatment [123,124,125,126,127].

Autologous dentin, obtained from extracted tooth elements, is configured as a fully biocompatible biomaterial of endogenous origin, reducing the risk of adverse immune responses or infection [128,129,130,131,132]. In addition, the particular consistency and cohesiveness of the resulting biomaterial facilitates its application and stabilization at recipient sites, contributing to improved healing time and quality of the regenerative outcome [133,134,135]. Clinically, the technique proves versatile and applicable in heterogeneous contexts, from single extraction cases to complex rehabilitations of entire arches [136,137,138]. Radiographic and clinical evidence supports the ability of the combined Tooth Transformer^®^ + CGFs protocol to generate bone tissue with the structural and functional characteristics necessary to sustain implant load over time, without significant volumetric loss or peri-implant complications [139,140,141]. However, some limitations of the study should be pointed out, including the absence of a control group and the small size of the cohort analyzed, which necessitate further investigations on larger samples and with controlled experimental designs [142,143,144].

Future studies may also further investigate the biological and molecular mechanisms underlying tissue regeneration induced by the combination of CGFs and demineralized dentin in order to fully understand its regenerative efficacy [145,146,147,148].

### 4.1. Clinical and Radiographic Evaluation of the Regenerative Protocol

The total implant survival recorded, together with the absence of intra- or postoperative adverse events, confirms the validity of the regenerative protocol employed. The mean vertical bone augmentation of approximately 8–10 mm was confirmed by CBCT analysis at 6 and 84 months, demonstrating the efficacy of the biomaterial derived from autologous dentin, enriched with CGFs, in promoting stable and durable bone regeneration [149,150,151,152,153,154,155,156]. The maintenance of bone volume over the long term is a remarkable finding, as it counteracts one of the main limitations of regenerative procedures: post-surgical bone resorption [157,158,159,160,161,162,163].

Densitometric measurements, with values between 700 and 850 HU, indicate the formation of mature bone, compatible with the presence of osteoinductive factors such as BMP-2 and TGF-β, in addition to platelet factors carried by CGFs [164,165,166,167,168,169]. Therefore, the synergy between an osteoinductive substrate such as demineralized dentin and the biological activity of CGFs allows not only adequate regenerated volume but also tissue quality suitable for implant support [170,171,172,173,174].

The use of autologous dentin offers numerous advantages over synthetic or heterologous biomaterials, including elimination of the risk of disease transmission and total biological compatibility [175,176,177]. The automated treatment process provided by the Tooth Transformer^®^ device ensures high standardization, safety and quality of the final material, which is rich in collagen and osteoinductive proteins [178,179,180,181]. When compared to other autologous regenerative approaches, such as platelet-rich fibrin (PRF) or bone marrow stem cell concentrates, autogenous dentin offers distinct advantages. PRF provides a fibrin matrix and growth factors but lacks a mineralized collagen scaffold, whereas dentin contributes both structural support and bioactive molecules such as BMPs and TGF-β. Bone marrow stem cells, although promising, require additional invasive harvesting procedures. In this context, dentin represents a readily available, fully autogenous, and biologically active option that may combine the benefits of scaffolding with growth factor release, potentially explaining the long-term stability observed in our cohort [14,17,33,37,81].

The association with CGFs, obtained by centrifugation of peripheral blood, allows a gradual release of growth factors essential for angiogenetic and regenerative processes [182,183,184,185,186,187,188]. The formation of “*sticky bone*” which has a cohesive, gel-like consistency, facilitates the adaptation of the material within the recipient site, improving its stability and increasing the likelihood of successful surgery, particularly in critical areas such as the maxillary sinus [189,190,191].

The average ISQ value detected at the time of functional loading (≥70) at 6 months and the maintenance of implant stability up to 84 months are indicative of effective and prolonged osseointegration over time [192,193,194,195,196,197,198]. The presence of ≥5 mm residual bone ridge was a favorable condition for primary stability, but the supply of regenerated bone tissue by the combined protocol allowed significant vertical augmentation, expanding the possibilities of rehabilitation even in patients with severe maxillary atrophy [199,200,201,202].

### 4.2. Considerations on Representative Clinical Cases

The reported illustrative clinical cases demonstrate the high adaptability and effectiveness of the regenerative protocol, which can be applied in varying clinical settings, from single extractions to total upper arch rehabilitations [203,204,205]. Three-dimensional clinical and radiographic evidence confirms the formation of bone tissue with both favorable quantitative and qualitative characteristics, with maintenance of peri-implant tissues in the absence of inflammatory signs or complications. None of the patients reported clinical or functional complications during follow-up. No signs of infection, persistent pain, implant mobility, or need for further regenerative treatment were observed.

Overall, the results obtained corroborate the efficacy of the regenerative protocol based on autologous dentin treated with Tooth Transformer^®^ and supported by CGFs, in the context of sinus lift. Such evidence is consistent with previous studies, such as the one conducted on 21 patients with an average follow-up of 63 months, which reported an average marginal bone loss of only 0.12 mm and 100% implant survival.

### 4.3. Limitations of the Study

A major limitation of this study is the absence of a control group or comparison cohorts treated with conventional bone grafts, which restricts the possibility of attributing the observed positive outcomes exclusively to the studied protocol. In addition, the sample size of 31 patients, although respectable for a long-term single-center study, remains relatively small and therefore limits the generalizability of the results. The retrospective nature of the investigation further prevented the use of inferential statistical analyses (such as survival curves or regression models), making the findings essentially descriptive and observational. These limitations indicate that the present results should be interpreted with caution, and confirmation through larger, controlled prospective studies is required. Finally, although the average follow-up duration of 84 months is a strength, further long-term monitoring with histological evaluations could provide a more comprehensive picture of bone quality maintenance and biological response at the regenerated sites

### 4.4. Future Objectives

The synergistic effect of dentin-derived BMPs and collagen with the angiogenic and mitogenic factors from CGFs likely underlies the stable bone regeneration observed. Future directions include controlled prospective studies to validate these findings, as well as randomized clinical trials comparing autogenous dentin with CGFs with other grafting materials, in order to confirm its long-term efficacy and safety.

## 5. Conclusions

The results obtained confirm the validity and efficacy of the regenerative protocol based on the combined use of demineralized autologous dentin, processed using a Tooth Transformer^®^ device, and concentrated growth factors (CGFs), applied at the same time as sinus lift surgery. Clinical-radiographic analysis, conducted on a sample of 31 patients and supported by CBCT imaging and clinical follow-ups up to 84 months, showed stable peri-implant bone volume and no significant bone resorption. All patients were able to benefit from fixed prosthetic loading within six months after surgery, with no evidence of postoperative complications or loss of implant fixtures. The 100% implant success rate testifies to the high predictability of the adopted protocol. These results suggest that the use of demineralized autologous dentin associated with CGFs is a clinically viable and less invasive alternative to traditional autologous bone harvesting techniques, especially in cases of severe maxillary atrophy, thus helping to reduce morbidity and surgical complexity.

## Data Availability

The data presented in this study are available on request from the corresponding author.

## Abbreviations

Abbreviation	Definition
AFG	Autologous Fibrin Glue
BMP-2	Bone morphogenetic protein
CBCT	Cone Beam Computed Tomography
CGF	Concentrated Growth Factors
IGF	Insulin-like growth factor
ISQ	Implant Stability Quotient
OPT	Orthopantomography
PDGF	Platelet-Derived Growth Factor
PRF	Platelet rich fibrin
PRP	Platelet rich plasma
TGF-β1	Transforming Growth Factor beta 1
VEGF	Vascular Endothelial Growth Factor
